# Commentary on “CTLA-4 Blockade Suppresses Progression of Residual Tumours after Insufficient RFA”

**DOI:** 10.1007/s00270-020-02564-9

**Published:** 2020-07-24

**Authors:** B. Geboers, M. R. Meijerink

**Affiliations:** Department of Radiology and Nuclear Medicine, Amsterdam University Medical Centers (location VUmc), de Boelelaan 1117, 1081 HV Amsterdam, The Netherlands

## Introduction

Image-guided tumour ablation has developed into a well-established treatment paradigm for solid cancers. As reflected by the increasing number of key publications in CVIR, several needle-guided thermal and non-thermal ablation techniques have evolved into reliable and widely adopted therapies in modern clinical oncology. Although complete tumour eradication has always been the primary objective, fundamental changes to this basic concept may be in the offing. Anecdotal reports of spontaneous regression of distant tumours following local tumour ablation (i.e. abscopal effect) triggered research of the underlying systemic immune mechanisms. This effort is gradually evolving into novel treatment approaches where focal tumour ablation is combined with passive or active immunotherapy.

Immunomodulating properties have been identified for all energy-based needle-guided ablative modalities [1]. Evidence that local ablation may lead to systemic tumour control through the priming and boosting of tumour-specific adaptive immunity, in effect resulting in in vivo vaccination, is found in several tumour types [2]. Conversely, clinical evidence for ablation-induced counteractive pro-oncogenic effects similarly exists and has been linked to aggressive tumour development and worse outcomes [3].

Local tumour progression (LTP) due to an incomplete ablation remains a major drawback of needle-guided tumour ablation. In this issue of CVIR, we are treated to an article by Zhang et al. [4] who were the first to investigate administration of CTLA-4 blockade after an ‘insufficient’ radiofrequency ablation (RFA) as potential solution for the development of LTP. The authors report the tumour response, histopathology, and survival of 40 mice with subcutaneously implanted HCCs treated with either (1) IgG control antibody, (2) insufficient RFA, (3) CTLA-4 blockade, or (4) insufficient RFA followed by CTLA-4 blockade. Only mice in the combination therapy group survived the follow-up period of 48 days (5 out of 8) and 3 out of 8 mice showed completely vanished residual tumours, while none in the other groups responded. Addition of anti-CTLA-4 to insufficient RFA significantly improved survival and suppressed residual tumour growth. Combined with the histopathological observation of significantly increased CD4 + T cell infiltration and IFN-Y concentrations, these results are indicative for the establishment of an effective adaptive systemic immune reaction. Tumour re-challenging confirmed that the augmentation of anti-tumour immunity has potential to become durable as 2 out of the 5 surviving mice showed complete tumour rejection.

However, some degree of reservation towards the results is required as the follow-up time was short, side effects of the combination therapy were not reported and, foremost, because animal results can be far-off translation into clinical practice. Furthermore, 2 of the surviving mice developed rapid and massive tumour growth after tumour re-challenging suggestive for latent pro-oncogenic effects.

In conclusion, this study supports the rationale to combine thermal ablation with checkpoint inhibition for HCCs. This proof of concept now warrants further investigation of the optimal dosage, timing, and sequencing of both therapies taking into account potential pro-oncogenic effects. If results can be clinically reproduced, the combination regimen may not only provide an alternative strategy to prevent LTP after HCC ablation, even a paradigm shift towards intentional incomplete ablations combined with immune enhancing drugs is conceivable. Interventional oncologists are well positioned in the centre of oncological care and, given the rise of immunotherapy, the integration of immuno-oncology in interventional oncology is inevitable, as stated in the Society of Interventional Oncology White Paper on the emerging role of immunotherapy in interventional oncology [5]. Considering their expanding role interventionists should guide this exciting revolution while acknowledging close collaborations with immune-oncologists and medical oncologists. If smart combinations with focal therapy truly prove to trigger systemic anti-tumour effects and excite durable responses, focal ablation may eventually provide the bridge between local and systemic treatment.
